# Automatic Histogram Specification for Glioma Grading Using Multicenter Data

**DOI:** 10.1155/2019/9414937

**Published:** 2019-12-18

**Authors:** Xi Chen, Yaping Wu, Guohua Zhao, Meiyun Wang, Wenyi Gao, Qian Zhang, Yusong Lin

**Affiliations:** ^1^School of Software, Zhengzhou University, Zhengzhou, Henan 450002, China; ^2^Collaborative Innovation Center for Internet Healthcare, Zhengzhou University, Zhengzhou, Henan 450052, China; ^3^Department of Radiology, Henan Provincial People's Hospital, Zhengzhou, Henan 450003, China; ^4^School of Computer Science, Zhongyuan University of Technology, Zhengzhou 450000, China

## Abstract

Multicenter sharing is an effective method to increase the data size for glioma research, but the data inconsistency among different institutions hindered the efficiency. This paper proposes a histogram specification with automatic selection of reference frames for magnetic resonance images to alleviate this problem (HSASR). The selection of reference frames is automatically performed by an optimized grid search strategy with coarse and fine search. The search range is firstly narrowed by coarse search of intraglioma samples, and then the suitable reference frame in histogram is selected by fine search within the sample selected by coarse search. Validation experiments are conducted on two datasets GliomaHPPH2018 and BraTS2017 to perform glioma grading. The results demonstrate the high performance of the proposed method. On the mixed dataset, the average AUC, accuracy, sensitivity, and specificity are 0.9786, 94.13%, 94.64%, and 93.00%, respectively. It is about 15% higher on all indicators compared with those without HSASR and has a slight advantage over the result of a manually selected reference frame by radiologists. Results show that our methods can effectively alleviate multicenter data inconsistencies and lift the performance of the prediction model.

## 1. Introduction

Glioma is a prevalent fatal brain disease and the most malignant, which accounts for approximately 24.7% of all primary brain and other central nervous system tumors and 74.6% of malignant tumors [1]. The World Health Organization's guidelines for glioma diagnosis and treatment are divided into four levels, namely, I–II and III–IV for low-grade glioma (LGG) and high-grade glioma (HGG) [2]. In clinical applications, biological behavior, treatment options, and prognoses of patients with glioma of different grades are clearly different. Therefore, the accurate preoperation grading of Glioma is important. Magnetic Resonance Imaging (MRI) is characterized by multidirectional tomography and multiparameter high-resolution soft-tissue imaging and is widely used to evaluate the tumor heterogeneity [3]. MRI is commonly used in glioma grading because it can accurately display the location and size, and it correlates well with histological characteristics. In medical research, high quality data is difficult to obtain in a single institution, so it needs to be shared through multicenter. However, the difference of multicentre data is a serious challenge.

**Figure 1:**
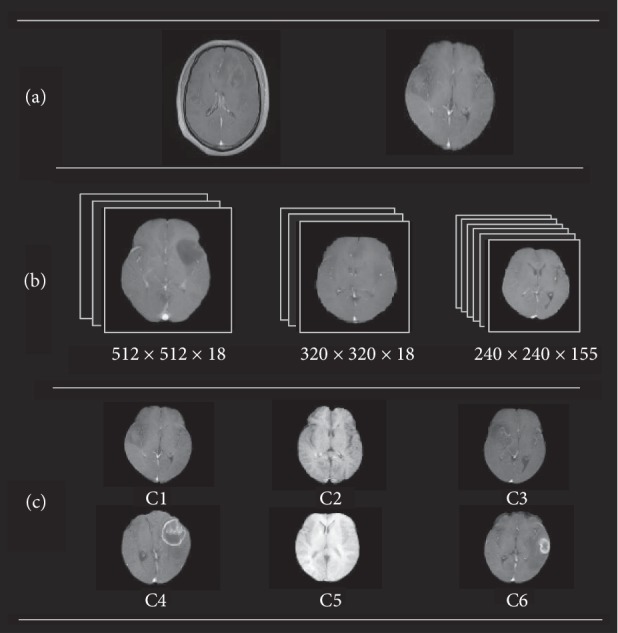
Multicenter data. (a) Space of dataset is uneven. (b) Volume pixel of data element is not uniform. (c) Dataset contrast brightness is not uniform, and C1–C6 are randomly selected data samples.

In the acquisition of multicenter glioma MRI data, due to differences in acquisition equipment and parameters result significant differences in data samples in terms of specification, size, contrast, and brightness. Differences among the data lead to deviations in the grading effect of glioma [4].  Figure 1 shows the differences among multicenter data.  Figure 1(a) shows that some of the data contain skulls and the others do not contain.  Figure 1(b) shows that these data have different scales and number of slices. A previous study indicated that pixel size and slice thickness remarkably affect space and strength characteristics of the calculation [5, 6]. Therefore, voxel size must be unified to reduce the error in calculating characteristics.  Figure 1(c) presents six images with different contrasts. These differences are due to deviation in acquisition equipment and parameters.

In order to alleviate the inconsistency of contrast of multicenter data, it is necessary to enhance the image data appropriately. Histogram correction is a commonly used technique in image enhancement [7]. Among them, histogram equalization (HE) and normalization techniques [8, 9] are often used to adjust the overall brightness of images. This type of methods mainly expands the range of gray value in the histogram without adjusting its intensity. These methods only enhance the overall image and improve its brightness, but the contrast between tumors and other tissues is not considerably enhanced [10, 11]. Histogram specification (HS) can change the frequency of grayscale values and enhance any local brightness [12, 13], but it mainly adjusts the local brightness according to the reference frame. In general, the reference frame is selected by a radiologist. However, this process consumes a considerable amount of time and the final reference frame may not be the best choice. Therefore, it is an urgent problem to automatically search for the best reference frame of histogram instead of searching by a radiologist.

This paper presents a histogram specification with automatic selection of reference frames for magnetic resonance images, called HSASR, specifically used to replace radiologists manually selecting reference frames. This method can enhance the consistency of tumor intensity in the whole medical image. In this work, we apply HSASR to the preprocessing of multicenter glioma data, and the effectiveness of this method is proved by Glioma grading experiment. The results showed that this method had certain practical value in glioma grading research.

The main contributions of this study are as follows:

1. This paper proposes a histogram specification with automatic selection of reference frames (HSASR). This method can automatically select the suitable reference frame of histogram instead of radiologists during image enhancement, so as to enhance the consistency of brain tumors image contrast.
2. This paper proposes a set of image standardization algorithms to make the preprocessed multicenter data have better consistency, improve the adaptability of data, and improve the accuracy of the glioma prediction model.

## 2. Related Works

In recent years, researchers have gradually adopted multicenter data to replace single institution in clinical medical research. However, multicenter data also faced many challenges. Berenguer et al. [14] carried out a test-retest phantom study of individual image acquisition parameters. The impact of image parameters on the image cannot be analyzed because of the difference of the model or machine manufacturer. Therefore, variations because of the use of images were eliminated by exclusively scanning phantoms. In addition, Hugo et al. [15] indicated that multicenter structure MRI studies have stronger statistical efficacy than single-institution studies. However, central differences in contrast sensitivity and spatial uniformity lead to differences in tissue classification or image registration that may reduce or completely offset the enhanced statistical efficacy of multicenter data. Therefore, maintaining data in a standard environment is important. Nyul et al. [16] mentioned some problems in the original MRI scale preprocessing method and attempted to use the median value and other percentile numerical methods, such as landmark, to solve these problems; they obtained robust results after preprocessing. The validity of the standardized new landmark was also mentioned in the study, after which the image brightness level and contrast consistency were significantly improved. Bakas et al. [17, 18] focused on the data preprocessing method, which has a certain enlightening effect on the preprocessing method of this study.

In preprocessing, contrast enhancement of multicenter data image is an important challenge. It was shown that a high-contrast medical image could lead to a better interpretation of the different adjacent tissues in the imaged body part [19, 20]. Accordingly, the resulting enhanced image, which is in terms of signal intensities of different tissues, can facilitate the automated segmentation, feature extraction, and classification of these tissues. Existing image enhancement techniques (empirical or heuristic) are remarkably related to a particular image and usually aimed at improving image contrast. However, no unified standard is available to measure the quality effect of image enhancement [21–24].

Image enhancement can be divided into two categories: spatial domain method and frequency domain method. Image enhancement in spatial domain usually corrects histogram. Histogram equalization is a commonly used global grayscale image enhancement technique, and the grayscale value is uniformly redistributed based on the cumulative density function of the histogram [25]. However, equalization fails to consider the intensity of the grayscale value. The average brightness of the image can make the regions with large original intensity fade, whereas dark areas will brighten after image equalization. Sengee et al. [26] suggested an extension method of BBHE based on the neighbourhood metric. This method involved a few steps: first, a large histogram was divided into the subregion using the neighbourhood metric and process independently. This method results in boundary noise and possibly uneven histogram brightness in two parts. In addition, MedGA [24] was an enhancement method that HE combined with genetic algorithm to directly improve the histogram frequency of images and has achieved obvious results. However, this method could only be limited to the presence of two grayscale regional tissues and not enhance complex brain tumors.

## 3. Materials and Methods

### 3.1. Data Collection

Glioma data used in the study were obtained from two separate sources, BraTS2017 and GliomaHPPH2018 datasets. The BraTS2017 dataset came from a variety of scanning instruments from 19 medical institutions. BraTS2017 dataset includes 210 HGG data and 75 LGG data; the data was already segmented when it was acquired. GliomaHPPH2018 dataset was obtained from multiple equipment of different models from multiple manufacturers, including 4 Siemens equipment (1 1.5t equipment, 3 3T equipment) and 4 GE equipment (2 1.5t equipment, 2 3T equipment), as shown in Table 1. The magnetic resonance data collected on these devices are different from each other. After data inspection, there are up to 186 default inspection protocols for the devices, and contrast-enhanced T1-weighted imaging (CET1) was used in this study, including 35 CET1 sequences (see Table 2), layer thickness of 5 mm to 6.5 mm, repetition time of 220–1970 ms, echo time of 2.46–28.60 ms, and resolution of 256 × 256 × 18, 320 × 290 × 18, 320 × 320 × 18, 384 × 384 × 18, 448 × 408 × 18, and 512 × 512 × 18. The GliomaHPPH2018 dataset includes 161HGG data and 77 LGG data. A three-dimensional region of interest (ROI) for all the tumors in GliomaHPPH2018 was depicted by two senior radiologists in Henan provincial people's hospital. The ROIs were segmented slice-by-slice on the axial plane using the CET1 sequences. After discussion between the two radiologists, the final decision is made to select the optimal segmentation file.

### 3.2. Histogram Specification

HS, an effective image enhancement technique, is an extension of histogram equalization that can effectively alleviate the problems of histogram equalization. Let *r* =  {*r*_*ij*_}be an *H* × *W* discrete input digital image with *L* gray levels, and let *L* = {0, 1,…, *L* − 1}. The histogram or gray level probability density of an image is defined as follows:

(1) $$\begin{array}{c} P_{r}\left(l\right)=\frac{N_{l}}{N},\;\forall l\in L, \end{array}$$

where *N* = *H* × *W* and *N* is the number of pixels with a gray level. The cumulative distribution function (CDF) of *P*_*r*_ is presented as follows:

(2) $$\begin{array}{c} s_{r}\left(x\right)=\overset{x}{\underset{j=0}{\sum }}P_{r}\left(j\right)=\overset{x}{\underset{j=0}{\sum }}\frac{N_{j}}{N},\;\forall x\in L. \end{array}$$

Similarly, if the specified reference image is *z*, then the gray cumulative distribution function is

(3) $$\begin{array}{c} v_{z}\left(y\right)=\overset{y}{\underset{i=0}{\sum }}P_{z}\left(i\right)=\overset{l}{\underset{i=0}{\sum }}\frac{M_{i}}{M},\;\forall y\in L, \end{array}$$

where *M* and *N* are the number of pixels with gray level *L*. HS attempts to obtain transformation function *y* = *F*(*x*) and maps gray level *x* in the original image to gray level *y*, such that the transformed image can have a histogram similar to the reference histogram. To preserve the inherent information of the original image, function *F* should be a monotonically increasing function. This function can be obtained by using the following equation:

(4) $$\begin{array}{c} v_{z}\left(y\right)=s_{r}\left(x\right). \end{array}$$

Therefore, the gray level of the map can be obtained by using

(5) $$\begin{array}{c} y=v_{z}^{-1}\left[s_{r}\left(x\right)\right], \end{array}$$

where *v*_*z*_^−1^ is the inverse of *v*_*z*_.

In the discrete case, the inverse function usually does not exist. The inverse function is usually replaced by the best objective function to approximate the *y* of a particular gray level *x* as follows:

(6) $$\begin{array}{c} y=\arg\underset{k}{\min}\;\left|\;s_{r}\left(x\right)-v_{z}\left(k\right)\right|. \end{array}$$

Equation (6) represents the absolute value of the difference between the original image's cumulative histogram and gray level functions of the specified reference histogram, and then the minimum value is selected as the *y* value. With this transformation rule, each gray level *x* can be mapped to *y*. Therefore, the mapped image will be similar to the desired histogram.

The reference frame in the histogram is usually selected by the doctor. However, the selected reference image may not be suitable, thereby consuming a considerable amount of the radiologist's time. To solve this problem, an optimized grid search strategy with coarse and fine search is proposed to select the suitable reference frame automatically.

### 3.3. HSASR

In this study, an improved grid search method is proposed to solve the problem of selecting a specified reference image. The grid search is generally used to divide grids of the same length in a certain spatial range based on the proposed coordinate system. Each coordinate point represents a parameter, and these searched parameters are calculated and analysed based on the step size. Finally, optimal parameters are considered the output. Given that this method must traverse all the corresponding points in the grid, many unnecessary invalid calculations are generated, resulting in an exponential increase in time.

To reduce time consumption, this study improves the directional grid optimization search based on data characteristics. On the basis of the characteristics of glioma itself, the method is improved by using coarse segmentation and subdivision. Horizontal and vertical searches are used for coarse segmentation and subdivision, respectively. The specific steps of the improved method are as follows:

1. The number of clusters *N* and the number of slices *K* of each tumor are determined. Each cluster is set to *i*, and the initialization step size is *K*/2. A 2D grid is established by using *N* and *K*, and the grid nodes are the corresponding reference slices of *N* and *K*.
2. In the coarse search, each time it searched by step size from the beginning of each set (horizontally), the median slice in each cluster was selected as the reference frame. Then, 30% of data in all datasets are randomly selected for histogram specification (the same sample is selected each time), and then the performance of data after histogram specification is tested.
3. Select the sets of all the best area under the curve (AUC) values according to threshold *T* and perform the next fine search.
4. Fine search: in the optimal cluster, the search is initiated from the middle section (two pointers are set to point to the middle section), and pointer 1 searches upward successively. If reference frames do not contain tumors, then the search is stopped. If the difference between the current and previous values is greater than *T*, then the search is stopped, and the optimal value is selected. At the same time, similar to the previous step, pointer 2 is searched downward.
5. The optimal reference slice map is the final output. If multiple optimal values exist, then selection is made based on accuracy and specificity indicators. If several optimal reference frames existed, then the first optimal reference frame is selected as the final reference frame.

**Figure 2:**
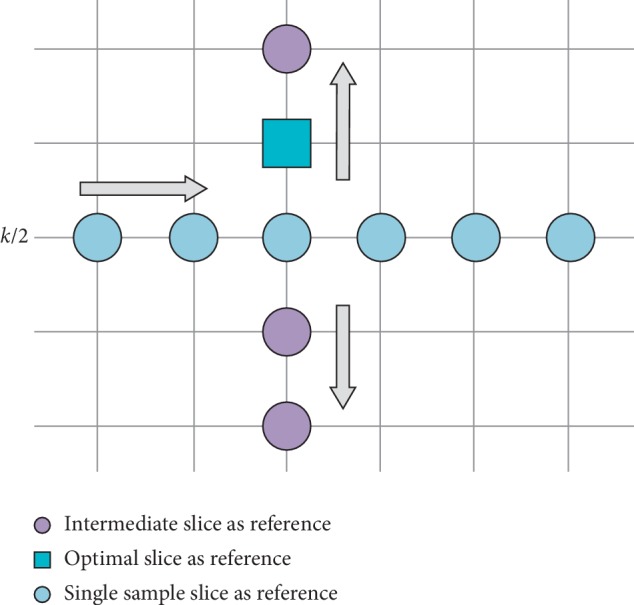
Diagram of the optimized grid search (note: *k*/2 represents the middle slice of the cluster).

Algorithm 1 describes the HSASR algorithm. The parameter *T* in Step 1 prevents the collection of additional optimal values from being missed. The *T* in Step 2 saves search time.  Figure 2 presents the 2D diagram of the improved optimization grid search. In this study, *N* represents the number of data samples, *K* denotes the number of slices of each sample, and *K*/2 is the middle slice of the sample. First, a horizontal coarse search is performed. The pale blue circles indicate the performance values of intermediate slices in each cluster. The optimal clusters are selected for longitudinal fine search, the middle slice is considered the initial value, and both ends are searched simultaneously. The vertical circles and dark blue squares represent the performance values in the optimal set and the selected final reference frame, respectively.

### 3.4. Experiments

**Figure 3:**
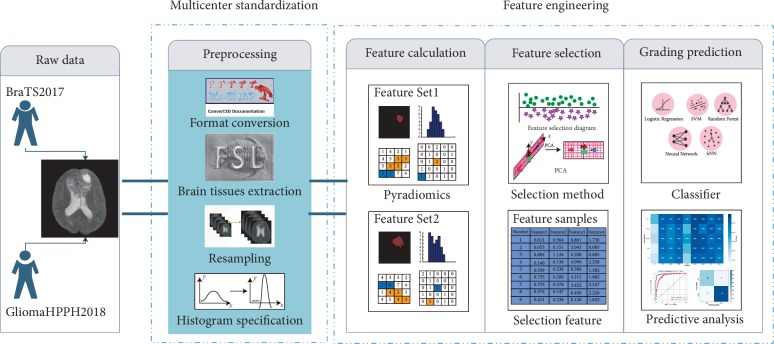
Flowchart of multicenter data grading prediction.

Figure 3 shows the overall process of multicenter data grading prediction. First, data are imported, data preprocessing is performed, features are extracted by using the feature calculation method, features are selected, and model training is finally conducted. Contribution points of this study are in the preprocessing stage. The HSASR method proposed is the most important part of preprocessing.

### 3.5. Preprocessing

Storage formats of multicenter data are often inconsistent. Data formats of this study include NIfTI and DICOM format files. Therefore, to standardize multicenter data formats, this study adopts the format conversion method based on the Convert3D tool to unify all data sample formats into the NIfTI format. The majority of patients in the GliomaHPPH2018 dataset have 18 DICOM files with CET1 sequence, and a small number of patients have 36 files. After format conversion, all slices of each patient's CET1 sequence are converted into the NIfTI format file, thereby providing convenience in subsequently unifying data processing.

Furthermore, to unify the multicenter data in preprocessing, maintaining the consistency of brain tissue structure is also important. The GliomaHPPH2018 dataset in this study contained skull images, which are removed at the time of acquisition in the BraTS2017 dataset. This situation is not only a certain impact on HS but also introduced difficulties in combining the two datasets due to the gap between them. In this study, the FSL tool is firstly used in location registration, and then the skulls are removed based on the bet script. In this study, 238 data skulls are removed, and brain tissues and regions of interest are relatively intact.

In addition, spatial consistency of the data image is maintained. Multiple voxel ranges occur in both datasets. On the basis of Convert3D, we adopt the shortest distance interpolation resampling technology to carry out scale resampling in each data sample. The size of each data sample is unified to 240 × 240 × 155, and the label sample is also converted into a file of the same size. In addition, the distance between unified slices is 1 mm, and the original position was [0, −239, 0].

To solve the problem of inconsistent brightness and contrast of multicenter data, this paper proposes a histogram specification with automatic selection of reference frames for magnetic resonance images. On the basis of the characteristics of glioma itself, this paper puts forward an optimized grid search strategy with coarse and fine search. First, in coarse search, all data samples are selected as the object of reference selection in this study. Each file is considered a collection, and the step size is set to the middle number of the collection (78), starting from the beginning of every collection each time. The middle slice (78) of each collection is used as the reference slice of HS, 30% of image samples are randomly selected from all data, and image enhancement is carried out via HS and subsequently imported into the model verification process of this study. *T* is set to 1%, and the set with the highest AUC value and AUC value error less than 1% is selected. Furthermore, the study carries out fine search, the step size is changed to 1, data are searched from the middle number upward and downward, and the search is over according to the end condition.

### 3.6. Feature Engineering

All the data being processed are imported into feature calculation. For each region of interest, 557 radiomics features are calculated through Pyradiomics (Pyradiomics is a tool for the computational characteristics of medical images). In this study, 9 spatial geometric features are included, which are only calculated in the original space. Eighteen first-order statistical features and 43 texture features are calculated in the original data and eight wavelet decomposition spaces, respectively. For all image histological features, model training is conducted directly after the processing of missing values.

The calculated features are imported into the model script of this study for prediction. The model script is divided into two parts, namely, feature selection and classifier. Five selection methods and nine classifiers are combined to set up the classifier, and the training and testing sets are imported into the model for prediction. For the designs of experiment 2 and 3 in the next section, 80% and 20% of data are selected from the data table as training and testing sets, respectively. The prediction is then repeated 10 times. The average value of final results is obtained, and the classifier and selection methods with the best results are finally selected with the rating device. The selection methods used in the study are SelectKBest (f_classif) for classifying the tag features between tasks for ANOVA *f* values, principal component analysis (PCA), kernel principal component analysis (KPCA), independent component correlation algorithm (ICA), and factor analysis (FA). The classifiers used in this study are decision tree (DT), random forest (RF), bagging (BAG), binary search tree (BSA), naive Bayes (NB), multilayer perception (MLP), support vector machine (SVM), logistic (LR), and k-nearest neighbour (KNN). For the two separate datasets in experiment 1, the stable training model is selected by using a tenfold cross-validation scheme, and then the grading prediction is finally performed.

### 3.7. Experimental Comparison Designs

To verify the effectiveness of processed multicenter data, this study proposed the following process designs:

1. Take the processed GliomaHPPH2018 and BraTS2017 datasets as the training and testing sets, respectively. Then, use the two unpreprocessed datasets as contrast experiments.
2. Mix together 521 data samples from GliomaHPPH2018 and BraTS2017, preprocess the data, carry out feature selection and model training, and analyze the results.
3. Use single sequences from the processed and unprocessed GliomaHPPH2018 and BraTS2017 as comparison experiment.
4. Make comparison experiments between this research method and common image enhancement methods on the mixed dataset. Finally, compare these results with the untreated data. In addition, to verify the effectiveness of HSASR, compare the research results with the reference diagram results selected by radiologists.

## 4. Results and Discussion

### 4.1. Analysis of Image Enhancement

**Figure 4:**
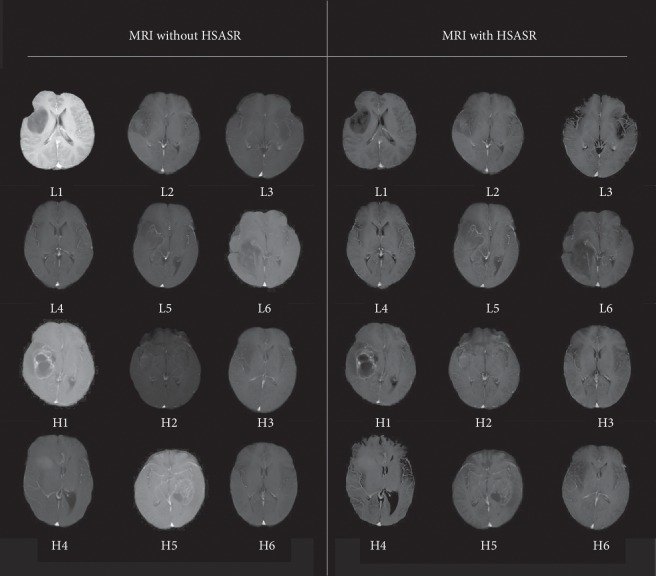
MRI comparison map before and after HSASR.

The purpose of weakening the multicenter data is to relieve the differences between groups and retain individual characteristics. That is to say, under the premise of retaining tumor morphological characteristics, the image data of different medical institutions have similar contrast and brightness. In this study, contrast and brightness of multicenter glioma data images are significantly improved after image enhancement in preprocessing.  Figure 4 shows the MRI results of the CET1 sequence in 12 patients. The left-hand side of Figure 4 presents that the first and second rows are LGG without HSASR, and the third and fourth rows are HGG without HSASR. All processed glioma data are shown on the right side of the figure. The examples show on the left and right sides had a one-to-one correspondence. From the perspective of image, the range of contrast and brightness of data without HSASR in Figure 4 are relatively complex and the tumor area is fuzzy and difficult to distinguish. The data after HSASR are consistent in contrast and brightness, the tumor area is significantly enhanced, and basic features (tumor morphology and lesion range) of the original image can be retained.

### 4.2. Performance of Glioma Grading

This paper aims to reflect the effectiveness of these methods indirectly by using model prediction indicators. To validate the effect of the processed multicenter data further, this section conducts the verification of grading results based on the following indicators: AUC, accuracy (ACC), sensitivity (SEN), and specificity (SPE).

**Figure 5:**
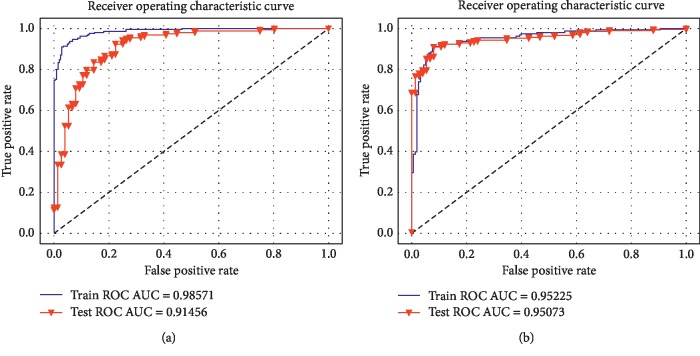
ROC plot.

Experiment 1 in Table 3 presents the results of GliomaHPPH2018and BraTS2017 datasets as the training and testing set, respectively. The results show that the data with HSASR is better predicted, and the indexes are significantly improved compared with the unprocessed data, as shown in Figure 5.  Figure 5(a) shows the ROC curve in which BraTS2017 is a training set and GliomaHPPH2018 is a testing set, and Figure 5(b) shows the ROC curve in which the testing set and the training set interchange. It can be observed from the figure that when testing set and training set swap, the better results can also be obtained. The results show that the data processed by the HRASR method have good adaptability and can alleviate the difference among multicenter data images.

Experiment 2 in Table 3 presents the results of the mixed dataset. The calculated characteristics are imported into the proposed grading model for 10 iterations of random prediction, and the average value is subsequently obtained. Finally, the model with the best average performance is selected as the final model. The results show that AUC after processing generally increased by more than 15%, and other indicators also improve significantly. Experiment 3 in Table 3 shows that the data processed by HSASR on a single dataset also achieve a good grading effect.

Experiment 4 in Table 4 shows the comparison experiment conducted by three different methods on the mixed dataset. These methods include the HE method, reference frame method of manual selection by radiologists, and the method proposed in this paper. The results show that compared with the other two methods, the method proposed in this paper significantly improves the grading effect of glioma. The reference frame selected by radiologists based on experience shows significant improvement in glioma grading, but it can be seen from the results that the performance of the selected reference frame is worser than that of the automatically selected.

Table 5 gives the performance comparison of similar work in the references using glioma dataset. In [27], after data preprocessing, they used a modified version of AlexNet for classifying MR brain images into three classes like healthy brain, LGG, and HGG. However, the amount of data of this work is so small that cannot verify the robustness of the proposed method. Reference [28] also involved data preprocessing, but our data is twice as large, and the result verified by the grading model is better than this method.

**Figure 6:**
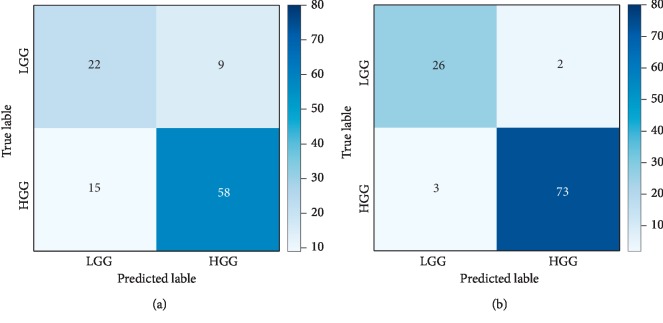
Confusion matrix of mixed dataset.

**Figure 7:**
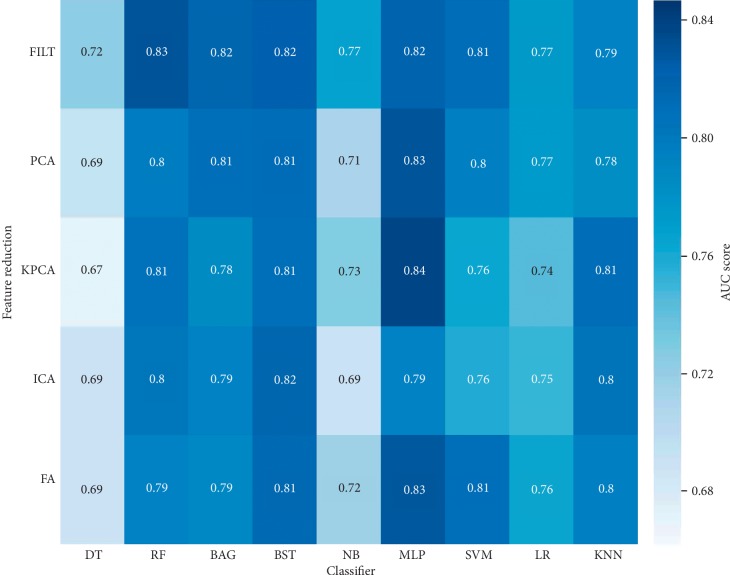
Heat map without HSASR.

**Figure 8:**
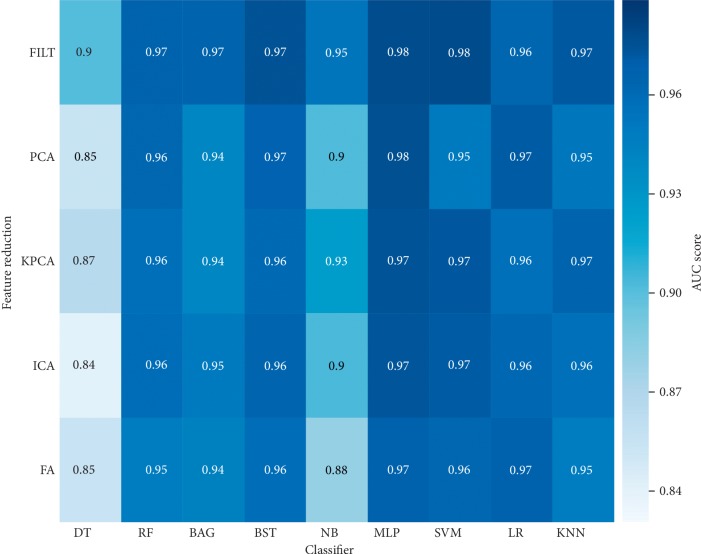
Heat map with HSASR.

Figure 6 is the confusion matrix corresponding to the final model of mixed glioma data in experiment 2, and LGG and HGG represent the low-level label and high-level label, respectively.  Figure 6(a) shows the confusion matrix before processing, and Figure 6(b) shows the predicted data distribution after processing. Figure 6 shows a significant decrease in the number of glioma predicted incorrectly after preprocessing. Meanwhile, in order to intuitively evaluate the performance differences of the model before and after preprocessing, Figures 7 and 8 list the performance heat maps of grading prediction before and after preprocessing, respectively. The horizontal and vertical coordinates and corresponding results represent the classifier, feature selection or dimension reduction method, and the maximum average AUC value, respectively. These data indicate that the overall performance of the data after preprocessing is about 15% higher than that without HSASR processing.

**Figure 9:**
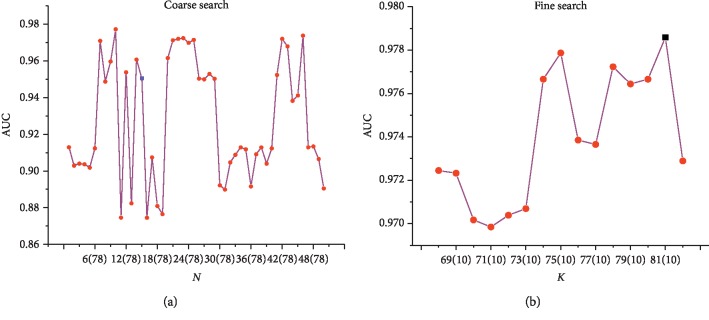
Diagram of the proposed method (Note: the red circle and blue square represent the AUC of machine and manually selected reference images, respectively. The black square represents optimal reference frame).

Figure 9 illustrates the experimental results of the grid search in this study. In this study, five reference slices with the highest performance indexes were selected from two datasets via rough search, and the reference slices with the best performance were screened out. The *N* axis, *K*, circle, and square denoted the number of samples, the number of slices in each sample set, the value of AUC of the reference image selected by the HSASR, and the predicted performance value of the reference image selected by the doctor, respectively. The obtained performance of the reference frame selected by the method is both low and high, indicating that the difference in reference objects had a significant impact on the result of grading. The proposed method can replace radiologists in choosing the best reference frame and save a considerable amount of time. Hence, the proposed method has certain application value in clinical trials.

## 5. Conclusions

Inconsistencies among data prevent multicenter data from playing to its shared advantage. This paper proposes a histogram specification method with automatic selection of reference frames for magnetic resonance images to alleviate the problem of contrast inconsistencies among multicenter data. The core of histogram specification is to change the local brightness of the image according to the reference frame, but the reference frame of traditional histogram specification is usually manually selected by radiologists. This method not only increases the workload of radiologists, but also cannot guarantee the optimal reference frame. In the method of this paper, the search range is firstly narrowed by coarse search intraglioma samples, then the suitable reference frame in histogram is selected by fine search within sample selected by coarse search. Finally, the effectiveness and feasibility of the proposed method are verified by a grading experiment based on two datasets. The results show that multicenter data processed by this method have good adaptability, which improves the grading results and has certain practical value for clinical prediction.

## Figures and Tables

**Algorithm 1:**
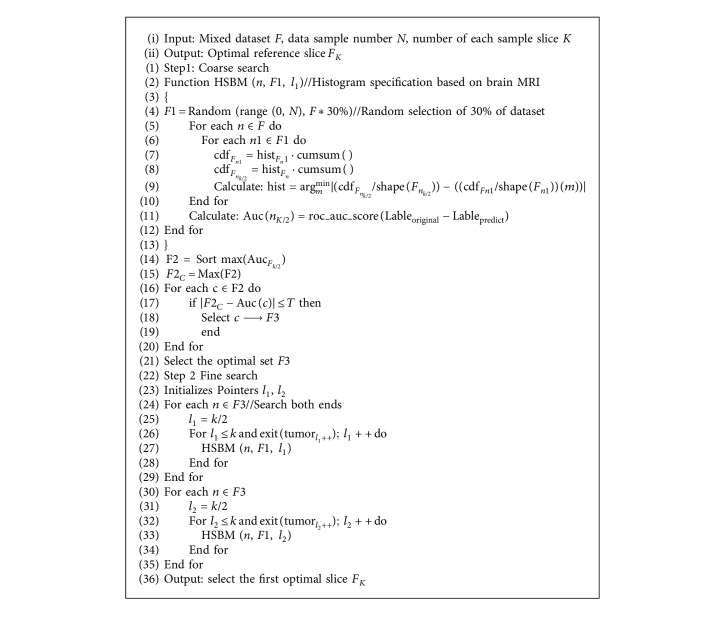
HSASR algorithm pseudocode description.

**Table 1:** GliomaHPPH2018 MR acquisition equipment list.

Number	Manufacturer	Model	Magnetic field strength	Amount
1	GE	OPTIMA MR360	1.5 T	1
2	GE	Signa HDxt	1.5 T	1
3	GE	DISCOVERY MR750	3 T	2
4	SIEMENS	Sempra	1.5 T	1
5	SIEMENS	Prisma	3 T	1
6	SIEMENS	TrioTim	3 T	1
7	SIEMENS	Verio	3 T	1

Note: amount is for number of machines.

**Table 2:** CET1 sequence collection parameter statistics.

Number	Manufacturer	Magnetic field	Model	*D*-spacing	Layer thickness	Resolution	Voxel size
1	GE	1.5	OPTIMA MR360	6.5	5.5	512 × 512 × 18	0.4688\0.4688
2	GE	1.5	OPTIMA MR360	7	6	512 × 512 × 18	0.4688\0.4688
3	GE	1.5	OPTIMA MR360	7.5	6	256 × 256 × 18	0.9375\0.9375
4	GE	1.5	OPTIMA MR360	7.5	6	512 × 512 × 18	0.4688\0.4688
5	GE	1.5	OPTIMA MR360	8	6	512 × 512 × 18	0.4688\0.4688
6	GE	1.5	Signa HDxt	7	6	512 × 512 × 18	0.4688\0.4688
7	GE	1.5	Signa HDxt	7	6	512 × 512 × 18	0.5273\0.5273
8	GE	1.5	Signa HDxt	7.5	6	512 × 512 × 18	0.4688\0.4688
9	GE	1.5	Signa HDxt	7.5	6.5	512 × 512 × 18	0.4688\0.4688
10	GE	1.5	Signa HDxt	8	6	512 × 512 × 18	0.4688\0.4688
11	GE	3	DISCOVERY MR750	6.5	5	512 × 512 × 18	0.4688\0.4688
12	GE	3	DISCOVERY MR750	7	6	512 × 512 × 18	0.4688\0.4688
13	GE	3	DISCOVERY MR750	7.5	6	512 × 512 × 18	0.4688\0.4688
14	SIEMENS	1.5	Sempra	7.2	6	448 × 408 × 18	0.5134\0.5134
15	SIEMENS	3	Prisma	7.2	6	320 × 320 × 18	0.7188\0.7188
16	SIEMENS	3	Prisma	7.2	6	384 × 384 × 18	0.5989\0.5989
17	SIEMENS	3	Prisma	7.8	6	320 × 320 × 18	0.7188\0.7188
18	SIEMENS	3	TrioTim	6.6	6	320 × 320 × 18	0.7188\0.7188
19	SIEMENS	3	TrioTim	6.6	6	320 × 320 × 18	0.75\0.75
20	SIEMENS	3	TrioTim	7.2	6	256 × 256 × 18	0.8984\0.8984
21	SIEMENS	3	TrioTim	7.2	6	256 × 256 × 18	0.9375\0.9375
22	SIEMENS	3	TrioTim	7.2	6	320 × 290 × 18	0.7188\0.7188
23	SIEMENS	3	TrioTim	7.2	6	320 × 320 × 18	0.7188\0.7188
24	SIEMENS	3	TrioTim	7.2	6	320 × 320 × 18	0.75\0.75

**Table 3:** Results of the first three glioma grading experiments.

Training	Testing	Without HSASR				With HSASR			
		AUC	ACC (%)	SEN (%)	SPE (%)	AUC	ACC (%)	SEN (%)	SPE (%)
GH	BT	0.6850	59.93	58.45	64.00	**0.9507**	**90.88**	**90.18**	**82.67**
BT	GH	0.7605	72.68	81.98	53.25	**0.9146**	**85.90**	**89.87**	**77.63**
(80%) (GH + BT) + (20%) (GH + BT)		0.8252	78.17	85.42	61.67	**0.9786**	**94.13**	**94.64**	**93.00**
(80%)GH + (20%)GH		0.8394	81.25	82.06	76.32	**0.9556**	**89.36**	**87.20**	**94.71**
(80%)BT + (20%)BT		0.8512	79.12	80.19	74.99	**0.9934**	**95.61**	**97.35**	**92.65**

Note: GH is for GliomaHPPH2018, and BT stands for BraTS2017. BT represents 80% of the data as training and 20% as testing. GH alone represents 80% of GH data as training and 20% of GH as testing. BT alone represents 80% of BT dataset as training and 20% of BT as testing.

**Table 4:** Results of the contrast experiment.

Training	Testing	Method	AUC	ACC (%)	SEN (%)	SPE (%)
(80%) (GH + BT) + (20%)	(GH + BT)	HE	0.9176	86.19	90.44	76.05
(80%) (GH + BT) + (20%)	(GH + BT)	Handpicked	0.9447	89.90	90.35	88.88
(80%) (GH + BT) + (20%)	(GH + BT)	Our method	**0.9786**	**94.13**	**94.64**	**93.00**

Note: GH is for GliomaHPPH2018, and BT is for BraTS2017. GH + BT represents 80% of the data as training and 20% as testing.

**Table 5:** Performance comparison with similar works.

Author and reference	Architecture/method	Output class	Data	Maximum ACC (%)
Khawaldeh et al. [27]	AlexNet + Preprocessing	3	130	91.16
Chen et al. [28]	CAD + Preprocessing	2	274	91.27
Our method	HSASR	2	523	94.13

## Data Availability

The datasets used in this paper are public dataset (BraTS2017) and Henan Provincial People's Hospital (GliomaHPPH2018) dataset; BraTS2017 can be obtained through the following URL: https://www.med.upenn.edu/sbia/brats2017/data.html.
